# Correlation between the optic nerve pial diameter and radial peripapillary vascular changes in primary open-angle glaucoma

**DOI:** 10.1007/s00417-021-05438-z

**Published:** 2021-10-21

**Authors:** Daniela Montorio, Gilda Cennamo, Maria Angelica Breve, Feliciana Menna, Michele Reibaldi, Giovanni Cennamo, Vincenzo Brescia Morra

**Affiliations:** 1 Department of Neurosciences, Reproductive Sciences and Dentistry, University of Naples Federico II, Naples, Italy; 2 Eye Clinic, Department of Public Health, University of Naples Federico II, Via S. Pansini 5, 80133 Naples, Italy; 3 Department of Ophthalmology, Ospedale Italiano Di Lugano, Lugano, Switzerland; 4 Department of Surgical Sciences, University of Torino, Torino, Italy

**Keywords:** Optic nerve pial diameter, A-scan ultrasound, Primary open angle glaucoma, Optical coherence tomography angiography

## Abstract

**Purpose:**

To assess the optic nerve pial diameter (ONPD) in patients with primary open-angle glaucoma (POAG) using standardized A-scan ultrasound and to evaluate the correlation between the ONPD and structural, vascular optic nerve head features and visual field parameters in glaucomatous eyes.

**Methods:**

In this prospective study, we enrolled 126 eyes of 63 POAG patients and 124 eyes of 62 healthy controls. In all subjects, the ONPD was evaluated by means of A-scan ultrasound. Spectral domain (SD)-OCT was used to assess ganglion cell complex (GCC), retinal nerve fiber layer (RNFL), thicknesses, and the optic nerve head (ONH) morphology. OCTA measured the vessel density (VD) of radial peripapillary capillary (RPC) plexus.

**Results:**

The ONPD showed a statistically significant reduction in POAG group with respect to controls (*p* < 0.001). SD-OCT and OCTA parameters showed a significant impairment in patient group with respect to controls (*p* < 0.001). The ONH analysis revealed significantly lower values in rim area (*p* = 0.009) and an increased cup-to-disc area ratio (*p* = 0.013) and cup volume (*p* < 0.001) in patients with respect to controls. Significant correlations were shown in POAG group between ONPD and RPC plexus (*p* = 0.006). Moreover, significant correlation was also found between ONPD and structural SD-OCT parameters (*p* = 0.001) and between ONPD and visual field parameters (*p* = 0.001).

**Conclusions:**

The standardized A-scan ultrasound measurements of the ONPD showed a significant correlation with structural and vascular glaucomatous changes measured by means of SD-OCT and OCTA. These results confirm the diagnostic reliability of the ultrasound evaluation in glaucoma optic neuropathy.

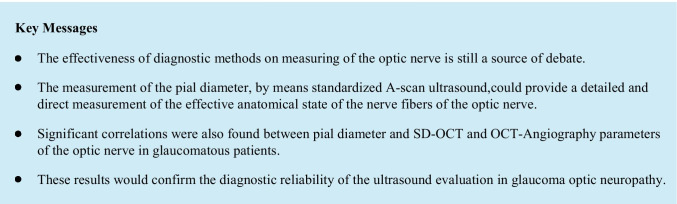

## Introduction

The evaluation of the anatomical structure of the optic nerve plays an important role in the diagnosis and follow-up of the glaucomatous optic neuropathy [1]

Several studies reported that the reduced optic nerve sheath diameter is strongly correlated with lower cerebrospinal fluid pressure (CSFP) that influenced, together with intraocular pressure, the translaminar pressure gradient involved in the pathogenesis and the progression of glaucoma [2–7].

The non-invasive evaluation of the optic nerve subarachnoid space by standardized A-scan ultrasound provided an indirect evaluation of the CSFP avoiding invasive method in clinical practice, such as the lumbar puncture [8].

Indeed, previous studies, demonstrating the close and direct relationship between any swelling of the optic nerve sheaths and the increase of the intracranial pressure, confirmed the validity and reliability of A-scan ultrasound [9, 10].

The effectiveness of diagnostic methods on measuring of the optic nerve is still a source of debate.

The diagnostic accuracy of B-scan ultrasonography and computed tomography or magnetic resonance imaging of the optic nerve sheath diameter do not always perform standard measurements, and they may be source of possible bias and errors of evaluation [11, 12].

Several studies focused on the evaluation of the changes in optic nerve subarachnoid space, but until now, no study have evaluated the pial sheath that represents the innermost membrane of the optic nerve, and the measurement of the pial diameter could allow a detailed and direct method to quantify the effective anatomical state of the nerve fibers of the optic nerve.

The purpose of this prospective study was to evaluate the optic nerve pial diameter (ONPD) by standardized A-scan ultrasound and to investigate the relationship between ONPD and structural and vascular changes of the optic nerve head by means of spectral domain optical coherence tomography (SD-OCT) and OCT-Angiography (OCTA) in early and moderate glaucomatous patients.

## Materials and methods

In this prospective study, one hundred twenty-six eyes of 63 patients presenting early to moderate primary open-angle glaucoma (POAG) and 124 eyes of 62 healthy subjects were enrolled from January 2019 to February 2020 at the Eye clinic of the University of Naples “Federico II.”

All subjects underwent a complete ophthalmological examination including the best-corrected visual acuity (BCVA) evaluation, intraocular pressure (IOP) with Goldman applanation tonometry, slit lamp biomicroscopy, gonioscopy, central corneal thickness, fundus examination with a + 90 D lens, standard visual field perimetry (Humphrey Field Analyzer with Swedish Interactive Thresholding Algorithm SITA standard 30–2 test program (Carl Zeiss Meditec Dublin, CA, USA), SD-OCT, OCT-A, and standardized A-scan ultrasound (Aviso S System, Quantel Medical, Bozeman, MT, USA).

Glaucomatous eyes presented visual field defects, optic nerve head changes such as localized rim thinning, increased cupping, and inter-eye cup asymmetry > 0.2 and open angle on gonioscopy. All patients, under topical medication, showed values of IOP within the normal range.

The healthy subjects that represented the control group included no family history of glaucoma or previous ocular diseases, unremarkable ophthalmic examination, IOP less than 21 mmHg, normal visual field test, and SD-OCT and OCTA parameters within normal limits.

Exclusion criteria were current, past ocular, or neurological diseases that could affect visual field and optic nerve function; history of intraocular surgery, congenital eye disorders, myopia greater than 6 diopters, uveitis, and diabetic retinopathy; previous cranial surgery or brain trauma; clinically relevant opacities of the optic media; and low-quality images obtained with SD-OCT and OCTA.

The study was approved by the Institutional Review Board of the University of Naples “Federico II” (protocol number: 142/19), and all investigations adhered to the tenets of the Declaration of Helsinki. Signed informed consents were obtained from each subject.

### A-scan ultrasound

The measurement of the pial diameter was determined by echographic examination using the standardized A-scan method as described by Ossoining and associates [13–18]. Topical anesthetic was instilled in the conjunctival sac of all participants in supine position before starting the exam. After application of gel on the probe, the A-scan probe was placed on the eye in the primary position at lateral canthus and was angled back at the orbital level of the optic nerve until the characteristic optic nerve pattern was obtained on the echographic screen. Then the optic nerve was examined when the patient rotates eye in lateral gaze position, at least 30° toward the probe (30° test) to contract and to stretch the elastic perineural sheaths redistributing the subarachnoid fluid and decreasing width of the space. The 30° test allows to evaluate reliably the ONPD without the interference of the subarachnoid space.

All measurements were performed at a distance of approximately 2 to 5 mm posterior to the globe. When the sound beam was perpendicular to the surface of the anterior third of the optic nerve, the highest double spikes that corresponded to pial surfaces were obtained from each side of the nerve.

By placing electronic gates over the peaks of the two maximized pial spikes, the maximum pial diameter was measured. All measurements were performed by the same experienced examiner not informed with glaucoma diagnosis.

### Spectral domain optical coherence tomography

The mean circumpapillary RNFL and GCC thickness were examined using SD-OCT (software RTVue XR version 2017.1.0.151, Optovue Inc., Fremont, CA, USA). The optic nerve head (ONH) analysis measured the rim area, disc area, cup-to-disc area ratio, and cup volume. Moreover, RNFL thickness was calculated along a 3.45-mm diameter circle around the optic disc [19].

The GCC thickness was obtained from a 7 × 7 mm grid of the macula centered 1-mm temporal to the fovea. The GCC thickness was analyzed from the internal limiting membrane to the outer boundary of the inner plexiform layer [20].

Only high-quality images with a signal strength index above 80 were accepted. The examiner rejected scans that had motion artefacts, incorrect segmentation, and poor centration and focus.

### Optical coherence tomography angiography

OCTA was performed by the Optovue Angiovue System (software ReVue XR version 2017.1.0.151, Optovue Inc., Fremont, CA, USA) that is based on a split-spectrum amplitude de-correlation algorithm (SSADA) and which uses blood flow as intrinsic contrast [21].

The AngioVue disc mode automatically calculated the VD of the radial peripapillary capillary plexus (RPC) that extended from the ILM to the RNFL posterior boundary. The VD was defined as the percentage area occupied by vessels in the analyzed region [22]. The VD measurements of the RPC were performed over a scan area of 4.5 × 4.5 mm centered on the optic disc (whole image) [23].

Images with a signal strength index less than 80, residual motion artefacts, incorrect segmentation, or low centration and focus were excluded.

### Statistical analysis

Statistical analysis was performed with the Statistical Package for Social Sciences (Version 20.0 for Windows; SPSS Inc., Chicago, IL, USA). We evaluated the differences between patients and controls in ONPD, as well as in structural OCT, OCTA, and visual field parameter changes through general linear models (GLM), including age and sex as covariates. Subject ID was included in all models as random factor to account for within-subject inter-eye correlation. The correlation between pial diameter values and OCTA, SD-OCT, and visual field parameters in POAG group was evaluated by Pearson’s correlation. The agreement of the intraobserver variability in the A-scan measurements was assessed using the intraclass correlation. A *p* value of < 0.05 was considered statistically significant.

## Results

A total of 126 eyes of 63 POAG patients (33 females, 30 males, mean age 70.14 ± 6.15 years) and 124 eyes of 62 healthy subjects (32 females, 30 males, mean age 69.22 ± 5.77 years) were included in this prospective study. There were no statistically significant differences for age (*p* = 0.392), sex (*p* = 0.655) and IOP measurements between the two study groups (*p* = 0.546) (Table 1).

**Table 1:** Demographic and clinical data of controls and primary open angle glaucoma patients

	**Controls**	**POAG**	***p*-value**
Eyes (*n*.)	124	126	-
Age (years)	69.22 ± 5.77	70.14 ± 6.15	0.392
Sex (female/male)	32/30	33/30	0.655†
IOP (mmHg)	15.22 ± 1.92	16.41 ± 1.52	0.546
CCT (µm)	542.23 ± 25.42	545.41 ± 23.11	0.752
MD (dB)	− 0.80 ± 1.41	− 5.48 ± 2.20	< 0.001
PSD (dB)	2.03 ± 0.91	5.32 ± 3.50	< 0.001

Data are expressed as mean ± SD

*POAG* primary open angle glaucoma, *IOP* intraocular pressure, *CCT* central corneal thickness, *MD* mean deviation, *PSD* pattern standard deviation, *dB* decibel

General linear models, statistical significance *p* value < 0.05

^†^Chi-squared test

At visual field, mean deviation values were − 5.48 ± 2.20 dB (range − 2.64 to − 8.28) in glaucomatous patients. They were divided, according to Hodapp-Anderson-Parrish criteria into 2 groups: early glaucoma (62 eyes) and moderate glaucoma (64 eyes).

The agreement of the intraobserver variability for measuring the A-scan parameter was excellent, with an intraclass correlation coefficient of > 0.8.

As shown in Table 2, ONPD values were significantly lower in POAG group with respect to controls (*p* < 0.001).

**Table 2:** Comparison of ONPD, OCTA, and structural SD-OCT parameters between controls and primary open angle glaucoma patients

	**Control group**	**POAG**	***p*-value**
ONPD (mm)	2.98 ± 0.13	2.62 ± 0.18	< 0.001
RPC plexus whole image	56.44 ± 4.58	42.87 ± 3.31	< 0.001
Ganglion cell complex (µm)			
*Average*	94.75 ± 7.11	77.16 ± 6.01	< 0.001
*Superior*	95.09 ± 7.19	79.75 ± 7.31	< 0.001
*Inferior*	93.80 ± 8.71	74.62 ± 7.25	0.001
Retinal nerve fiber layer (µm)			
*Average*	101.11 ± 7.83	84.55 ± 7.09	< 0.001
*Superior*	102.21 ± 8.94	87.66 ± 8.53	< 0.001
*Inferior*	100.73 ± 8.80	79.64 ± 8.30	< 0.001
Optic nerve head analysis			
*Rim area (mm*^*2*^*)*	1.45 ± 0.38	0.81 ± 0.27	0.002
*Disc area (mm*^*2*^*)*	2.47 ± 0.41	2.49 ± 0.61	0.995
*Cup-to-disc area ratio*	0.41 ± 0.25	0.65 ± 0.19	0.013
*Cup volume (mm*^*3*^*)*	0.057 ± 0.51	0.532 ± 0.46	< 0.001

Data are expressed as mean ± SD

*POAG* primary open angle glaucoma, *ONPD* optic nerve pial diameter, *RPC* radial peripapillary capillary

General linear models, statistical significance *p* value < 0.05

At OCTA examination, the VD of RPC was significantly impaired in patients with respect to healthy subjects (*p* < 0.001).

The structural SD-OCT parameters showed a statistically significant reduction in GCC parameters in POAG patients with respect to controls (*p* < 0.001), as well as in RNFL parameters (*p* < 0.001).

**Fig. 1:**
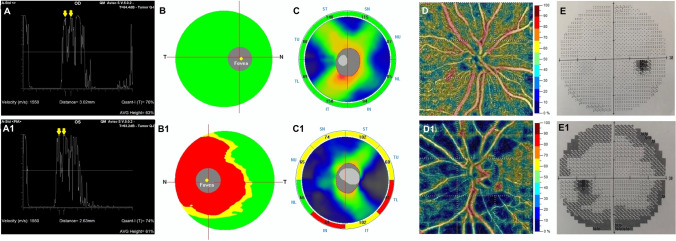
Right eye of a healthy subject (66-year-old female) shows normal values of optic nerve pial diameter (ONPD) at standardized A-scan ultrasound. The yellow arrows over the peaks of the two high and perpendicular spikes, as shown in echogram, indicate the maximum pial diameter measured in the anterior third of the optic nerve (A). Structural spectral domain (SD)–optical coherence tomography (OCT) revealed normal ganglion cell complex (GCC) (B) and retinal nerve fiber layer (RNFL) thicknesses (C). At OCT-Angiography (OCTA), the vessel density of radial peripapillary capillary (RPC) plexus does not show reduction (D), as well as there is no visual field loss (E). Left eye of a patient (68-year-old female) with primary open angle glaucoma reveals a reduction of ONPD at standardized A-scan ultrasound examination (yellow arrows shown in A1). Structural SD-OCT (B1, C1) parameters show a widespread damage at GCC and RNFL thicknesses. OCTA (D1) examination presents significant impairment in vessel density of RPC plexus. Peripheral visual field loss is present with respect to healthy subject (E1)

The ONH analysis revealed in patients a thinner rim area (*p* = 0.002), an increased cup volume (*p* < 0.001), and cup-to-disc area ratio (*p* = 0.013) with respect to controls (Fig. 1).

ONPD significantly correlated with RPC plexus (*p* = 0.006) and with GCC and RNFL average (*p* = 0.001 and *p* = 0.001, respectively).

ONH parameters, in particular rim area, cup-to-disc area ratio, and cup volume, showed statistically significant correlations with ONPD (*p* < 0.05). Significant correlations were also found between ONPD and visual field parameters (*p* = 0.001) (Table 3).

**Table 3:** Pearson correlation coefficient between ONPD and OCTA, SD-OCT, and visual field parameters in primary open angle glaucoma patients

	**ONPD**	
	***r***	***p*-value**
**RPC**	0.626	0.006
**GCC average**	0.531	0.001
**RNFL average**	0.512	0.001
**MD**	0.449	0.001
**PSD**	− 0.305	0.001
**Rim area**	0.401	0.001
**Disc area**	0.121	0.657
**Cup-to-disc area ratio**	− 0.522	0.014
**Cup volume**	− 0.426	0.023

*ONPD* optic nerve pial diameter, *RPC* radial peripapillary capillary, *GCC* ganglion cell complex, *RNFL* retinal nerve fiber layer, *MD* mean deviation, *PSD* pattern standard deviation

*R* Pearson’s correlation coefficient

Statistical significance *p* < 0.05

## Discussion

To our knowledge, this is the first study to measure the ONPD, using standardized A-Scan ultrasound, in patients affected by POAG in early and moderate stages.

Our results showed a statistically significant reduction of ONPD with respect to controls that could be explained by the vascular impairment of the optic nerve, confirmed by OCTA examination. Also SD-OCT parameters revealed a structural damage of the optic nerve head showing a significant thinning of the GCC, RNFL, rim area, and an increased cup-to-disc area ratio and cup volume.

Previous histological studies, as that conducted by Pache et al., have focused on the morphological changes in retrobulbar optic nerve in glaucoma highlighting a significant reduction in mean diameter due to the advanced loss of axons [24].

We also found that the reduced ONPD significantly correlated with the thinning of GCC, RNFL, and rim area and with the increased cup-to-disc area ratio and cup volume, as demonstrated by Jonas et al. that found a significant relationship between reduced optic nerve fiber count and impaired diameter of the optic nerve cross section after enucleation in glaucomatous patients [25].

Moreover, we demonstrated a significant association between lower ONPD and peripapillary vascular network loss confirming the crucial vascular role in the etiopathogenesis of glaucoma.

According to the vascular theory, the optic nerve blood flow damage due to both increased IOP and vascular risk factors (vascular dysregulation, local vasospasm, and nocturnal hypotension) could lead an axonal ischemia, as well as demonstrated by reduced RPC at OCTA in our results, determining an impairment of GCC and RNFL thicknesses with consequent ONPD reduction [26–33].

Our findings confirmed previous results shown by Dichtl et al. who were pioneers in the evaluation of a significant relationship in glaucomatous eyes between the thinning of pial diameter, evaluated at standardized A-scan ultrasound, and the narrowing of the retinal arterioles diameter at the optic disc border, the reduction of RNFL, and neuroretinal rim area, measured by means red-free photographs [34].

Since the close neurostructural and vascular interconnection between retina and central nervous system [35, 36], the use of standardized A-scan ultrasound together with SD-OCT and OCTA could demonstrate the relationship between anatomical damages of the optic nerve and the neurodegenerative processes involved in glaucoma.

The glaucomatous optic neuropathy, like many optic nerve diseases, is an example of how ocular echography is until today a valid method and an indispensable diagnostic technique in the ophthalmic field, despite the advent of new diagnostics methods.

This study evaluated the effect of the early and moderate glaucomatous changes in SD-OCT and OCTA parameters on the anatomical features of the optic nerve by measuring the pial diameter.

Our results showed that pial diameter changes are in relation with the alterations in structural and vascular findings of the optic nerve. Therefore, very slight impairment of SD-OCT and OCTA parameters in glaucomatous patients may determine a thinning of the ONPD.

Thus, it is evident that A-scan ultrasound examination of the ONPD provides an immediate evaluation, in real time, of the structural and vascular damages of the optic nerve, already in early and moderate glaucoma. This may be helpful for a reliable differential diagnosis in several pathologies of optic nerve lesions, such as glaucoma, inflammatory, and tumor diseases of the optic nerve.

In conclusion, standardized A-scan ultrasound provided a reliable and objective examination of the ONPD obtaining useful information regarding axonal damage, supported by the structural and vascular impairment of the optic nerve at SD-OCT and OCTA. Further longitudinal studies with larger sample are needed to better confirm these findings in different stages of glaucoma disease.
